# Solasonine Causes Redox Imbalance and Mitochondrial Oxidative Stress of Ferroptosis in Lung Adenocarcinoma

**DOI:** 10.3389/fonc.2022.874900

**Published:** 2022-05-18

**Authors:** Yao-Ying Zeng, Ying-Bin Luo, Xu-Dong Ju, Bo Zhang, Ya-Jing Cui, Yan-Bin Pan, Jian-Hui Tian, Wen-Jing Teng, Jianchun Wu, Yan Li

**Affiliations:** ^1^ Department of Oncology, Shanghai Municipal Hospital of Traditional Chinese Medicine, Shanghai University of Traditional Chinese Medicine, Shanghai, China; ^2^ Department of Respiratory Medicine, Shanghai Municipal Hospital of Traditional Chinese Medicine, Shanghai University of Traditional Chinese Medicine, Shanghai, China

**Keywords:** solasonine, ferroptosis, lung adenocarcinoma, oxidation, mitochondrial dysfunction

## Abstract

Ferroptosis, a type of iron-dependent oxidative cell death caused by excessive lipid peroxidation, is emerging as a promising cancer therapeutic strategy. Solasonine has been reported as a potential compound in tumor suppression, which is closely linked to ferroptosis. However, ferroptosis caused by solasonine is insufficiently identified and elaborated in lung adenocarcinoma, a fatal disease with high morbidity and mortality rates. First, the biochemical and morphological changes in Calu-1 and A549 cells exposed to solasonine are observed using a cell death assay and a microscope. The cell viability assay is performed after determining the executive concentration of solasonine to assess the effects of solasonine on tumor growth in Calu-1 and A549 cells. The ferroptosis is then identified by using ferroptosis-related reagents on CCK-8, lipid peroxidation assessment, Fe^2+^, and ROS detection. Furthermore, the antioxidant system, which includes GSH, Cys, GPx4, SLC7A11, and mitochondrial function, is measured to identify the potential pathways. According to the results, solasonine precisely exerts antitumor ability in lung adenocarcinoma cells. Ferroptosis is involved in the solasonine-induced cell death, as well as the accumulation of lipid peroxide, Fe^2+^, and ROS. Moreover, the failures of antioxidant defense and mitochondrial damage are considered to make a significant contribution to the occurrence of ferroptosis caused by solasonine. The study describes the potential process of ferroptosis caused by solasonine when dealing with lung adenocarcinoma. This encouraging evidence suggests that solasonine may be useful in the treatment of lung cancer.

## Introduction

It is common knowledge that when a cell proliferates at an uncontrollable rate with an abnormal shape and malfunctions, trouble is on the way. Cancer is a multistage disease that is driven by genetic factors, immunological problems, adaptive metabolism, and other mutations. When a process occurs in the lungs, it may indicate that lung cancer, a lethal tumor with the highest morbidity and fatality rates in the world, is posing a threat to your life. According to statistics, lung adenocarcinoma (LUAD), the most common subtype of lung cancer, accounts for approximately 40% of all lung cancer occurrences (1). Despite a slew of obstacles, researchers continue to push forward in oncology research. Regulated cell death (RCD) has recently become a primary focus of researchers because of its possible therapeutic value in cancer. Ferroptosis, a new type of cell death with specific features, could be an adaptive mechanism that plays a key part in the eradication of cancerous cells (2).

Ferroptosis, formally proposed in 2012, was characterized by iron overload and lipid reactive oxygen species accumulation in the biochemical process (3). Other distinctive traits included cytological abnormalities in mitochondria, which manifested as smaller mitochondria with no cristae, a high density of mitochondrial membrane, and even rupture of the outer mitochondrial membrane (4). Iron toxicity, antioxidant defense failure, free radical production, and mitochondrial fatty-acid metabolism were identified as pathways in the process of ferroptosis, and those factors required for ferroptosis were integrated into attenuating the selective permeability of the plasma membrane, definitively causing cell termination (5). Ferroptosis was discovered to have benefits in cancer treatment by reversing drug resistance, sensitizing radiation, and synergizing immunotherapy, along with related basic research rapidly developing (6). According to emerging investigations, many natural compounds with anti-tumor potential have been discovered by inducing ferroptosis.

Solasonine (SS), a natural glycoalkaloid compound, may be a promising candidate for cancer treatment development and advancement. SS has been demonstrated to be toxic to a variety of cancer cell lines, including hepatocellular carcinoma cells (7), lung cancer cells (8), acute monocytic leukemic cell lines (9), glioma cells (10), and gastric cancer cells (11). Besides apoptosis (7–9), cell cycle arrest (9) and anti-inflammatory (10), ferroptosis (12) was discovered to contribute to the pharmacological advantages of SS. However, the pharmacological mechanisms by which SS causes ferroptosis in LUAD have yet to be identified and interpreted.

We investigated the toxicity of SS on Calu-1 and A549 cells in this study. Afterward, the induction of ferroptosis in response to SS was confirmed by combining the detection of lipid ROS, Fe^2+^, and ROS with the application of multiple ferroptosis-related reagents. In addition to redox imbalance, mitochondrial oxidative stress was observed in cells exposed to SS in the following investigation. The findings of this study are intended to supplement the knowledge of SS in the application of cancer treatment.

## Materials and Methods

### Cell Culture and Treatment

Calu-1 and A549 cancer cells were obtained from the National Collection of Authenticated Cell Cultures (NCACC) and cultured in DMED medium supplemented with 10% fetal bovine serum (FBS) (Gibco) and 1% antibiotic in a humidified incubator at 37˚C and 5% CO_2_. Cells with a density of 70% to 80% were deemed ready to adopt measures for subsequent detection. To enhance the effect of the inhibitors used in this study, they were intentionally added to cells 2 h before SS.

### Reagents

The reagents included solasonine (HY-N0070, MCE), Z-VAD-FMK (S7023, Selleck), DCFH-DA (S0033S, Beyotime), Cell Counting Kit-8 (40203ES76, YEASEN), SYTOX green (S7020, Invitrogen), Trolox (C3183, APExBIO), DFO (HY-B0988, MCE), Mito-TEMPO (HY-112879, MCE), Necrostatin-1(HY-15760, MCE), Hoechst 3342 (23491-52-3, Solarbio), Ferrostatin-1 (HY-100579, MCE), RSL3 (HY-100218A, MCE), Bafilomycin A1 (HY-100558, MCE), C11-BODIPY581/591 (D3861, Invitrogen), Erastin (HY-15763,MCE), GSH assay kit (A006-2-1, Nanjing Jiancheng), Cysteine assay kit (BC185, Solarbio), FerroOrange probes (M489, Dojindo), MitoPeDPP (M466-5,Dojindo), and JC-1 (MT09-1, Dojindo).The following antibody purchased from Cell Signaling Technology was GAPDH (5174, CST), the others purchased from BOSTER included GPX4 (BM5231, BOSTER) and SLCA711 (BM5318, BOSTER). According to the manufacturer’s instructions, the drugs were initially dissolved in dimethylsulfoxide (DMSO) at a stock solution.

### Cell Viability Assay

Approximately 5000 Calu-1 cells and 10,000 A549 cells were seeded into the 96-well plates in triplicate and cultured in the appropriate conditions for 24 h. Incidentally, the objective drugs and reagents involving various inducers and inhibitors were applied based on the manufacturer’s protocol. Specifically, the inhibitors including Z-VAD-FMK (Z-V), Necrostatin-1 (Nec), Ferrostatin-1 (Fer-1), Bafilomycin A1 (Baf-A1), Deferoxamine mesylate (DFO), Mito TEMPO, Trolox, and RSL3 were added to the cells for 2 h prior to SS for pretreatment. The medium was then supplemented with the Cell Counting Kit 8 (CCK8) reagent, and the cells were incubated for 2 h. Finally, the absorbance at 450 nm was measured using a microplate reader (Bio Tek, United States).

### Cell Death Assessment

The SYTOX Green staining solution was used to visualize cancer cell death. Calu-1 and A549 cells were seeded into 24-well plates at a density of approximately 5*104 cells/well and 1*105 cells/well, respectively, and cultured at 37°C in an incubator. Calu-1 cells were exposed to SS (10 μM, 15 μM, 20 μM) after 24 h, while A549 cells were exposed to SS (20 μM, 25 μM, 30 μM). Meanwhile, the cells were stained with 100 nM SYTOX Green and cultured in the dark before being observed under a fluorescence microscope (Leica, Germany).

### Cellular Iron Detection

The levels of intracellular Fe^2+^ were assessed using FerroOrange probes (Dojindo) according to the manufacturer’s protocol. Cells were drug-treated for the time indicated before being stained with a final concentration of 1 mol/L of FerroOrange for 30 min at 37°C. The signal from the samples was collected using a flow cytometer and analyzed using the GraphPad Prism software.

### Measurement of Cytosolic Reactive Oxygen Species (ROS) Generation

In the study, DCFH-DA, a cell-permeable probe, was used to measure the levels of cytosolic ROS using flow cytometry and a fluorescence microscope. In brief, cells were drug-treated before being cultured with DCFH-DA for 30 min at 37°C. The level of ROS was measured using the flow cytometer and fluorescence microscope mentioned above after the samples were washed twice with PBS.

### Lipid Peroxidation Assessed by C11-BODIPY581/591

C11-BODIPY581/591 was performed on cells by incubating them in PBS containing 5% FBS and 10 μM C11-BODIPY581/591 for 1 h at 37°C in the dark after being treated as indicated. The cells were then rinsed twice with PBS and examined under a fluorescence microscope. The labeled cells were distinguished by a shift in the fluorescence emission peak from 590 nm to -510 nm, which was proportional to lipid ROS generation and would be measured using a flow cytometer.

### Determination of GSH Activity

The glutathione (GSH) concentration was determined using a glutathione assay kit purchased from Jiancheng Bioengineering institute and carried out according to the manufacturer s instructions. The treated cells were harvested and homogenized in PBS on ice, the detected result was read by a microplate reader, and the glutathione in the cell lysate was calculated using the formula provided by the GSH assay kit’s product protocol.

### Measurement of Intracellular Cysteine

Intracellular cysteine (Cys) levels were determined in the lysates of cells exposed to drugs using a Cys assay kit (BC185, Solarbio) according to the manufacturer’s instructions. The final data obtained from a microplate reader at an absorbance of 600 nm was calculated using the formula provided by the Cys assay kit’s product protocol.

### Mitochondrial Injury Assessment

The change in mitochondrial membrane potential was measured using JC-1 (Dojindo) staining as directed by the manufacturer. The ratio of red fluorescence to green fluorescence was used to describe each group.

### Mitochondrial Lipid Peroxidation

The MitoPeDPP **(**Dojindo), a cell-membrane-permeable probe that specifically localizes in mitochondria due to the triphenylphosphonium moiety, will be used to assess lipid peroxidation in mitochondria. Cells were treated as indicated, then washed twice with PBS and stained with 1 μM MitoPeDPP and 10 μM Hoechst nuclear stain in certain experiments. Following that, images of cells were captured at random by a fluorescence microscope with 100 m scale bars.

### Western Blot Assays

Total proteins were isolated using a cell lysis buffer supplemented with protease and phosphatase inhibitors (Beyotime, Shanghai, China). Protein concentrations were measured using a BCA protein assay kit (Beyotime). After boiling in the 1% SDS loading buffer, the protein samples were separated by SDS/PAGE and transferred to polyvinylidene fluoride (PVDF) membranes (Millipore, Billerica, MA). The samples were then incubated with the primary antibodies (GAPDH, SLC7A11, GPX4) overnight before being incubated with the secondary antibody anti-rabbit IgG (925-32211, LICOR) with the membranes. Finally, protein brand images were obtained using an infrared laser two-color image analysis system (Odyssey LICOR, United States). Image J was used to calculate and normalize the objective protein expression level.

### Data Analysis

In the study, all results obtained from experiments were independently repeated three times and tested for normality. The continuous variables of normal distribution or nearly normal distribution were displayed as average value ± standard deviation. After homogeneity of variance test, one-way ANOVA and LSD *post hoc* comparisons were used for those meeting data requirements in SPSS 25 or GraphPad Prism 8.0. P < 0.05 was considered significantly different.

## Results

### Solasonine Promoted Cell Death in LUAD Cells

**Figure 1:**
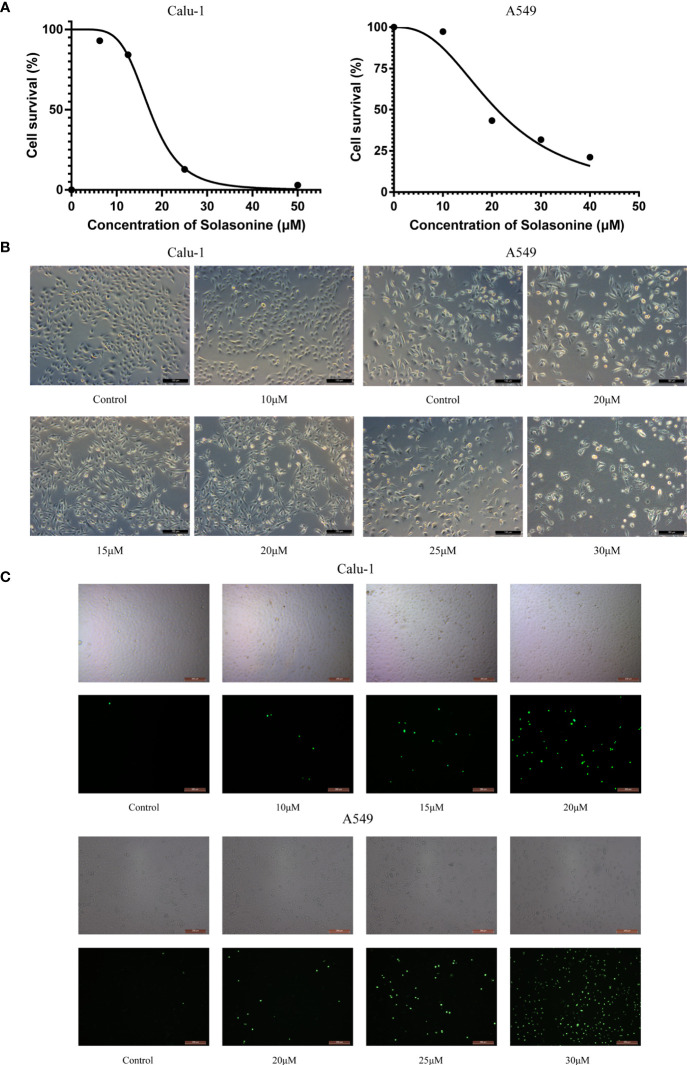
The cytotoxicity of solasonine in LUAD cells. **(A)** The cell viability of Calu-1 and A549 cells was measured using the CCK-8 assay after SS treatment for 24 h. **(B)** Microscope observation of Calu-1 or A549 cells treated with 10, 15, 20 μM or 20, 25, 30 μM of SS for 24 h, respectively. Scale bars: 100 μm. **(C)** Cells were exposed to different concentrations of SS and 100 nM of SYTOX Green for 24 h. The signal was captured and examined through the FITC channel of fluorescence microscopy. Scale bars: 200 μm.

Previous studies have demonstrated that SS has anti-proliferative properties. The Calu-1 and A549 cells, which belong to the LUAD cell lines, were used in this study for cytotoxicity experiments and subsequent examinations. The cells were subjected to the treatments described above, and the IC50 values of SS for Calu-1 or A549 cells were determined to be 15.08 or 21.59 μM, respectively (**Figure 1A**). Based on the findings, Calu-1 cells were found to be more sensitive to SS than A549 cells, so we tailored the appropriate drug concentrations for follow-up work. In addition, the cell morphology changed, with the cells becoming shriveled and even broken as the administration concentration increased (**Figure 1B**). Likewise, the fluorescent images indicated by the SYTOX Green confirmed that SS elicited cell death in a dose-dependent manner (**Figure 1C**).

### Ferroptosis Contributed to Solasonine-Induced Growth Inhibition in LUAD Cells

**Figure 2:**
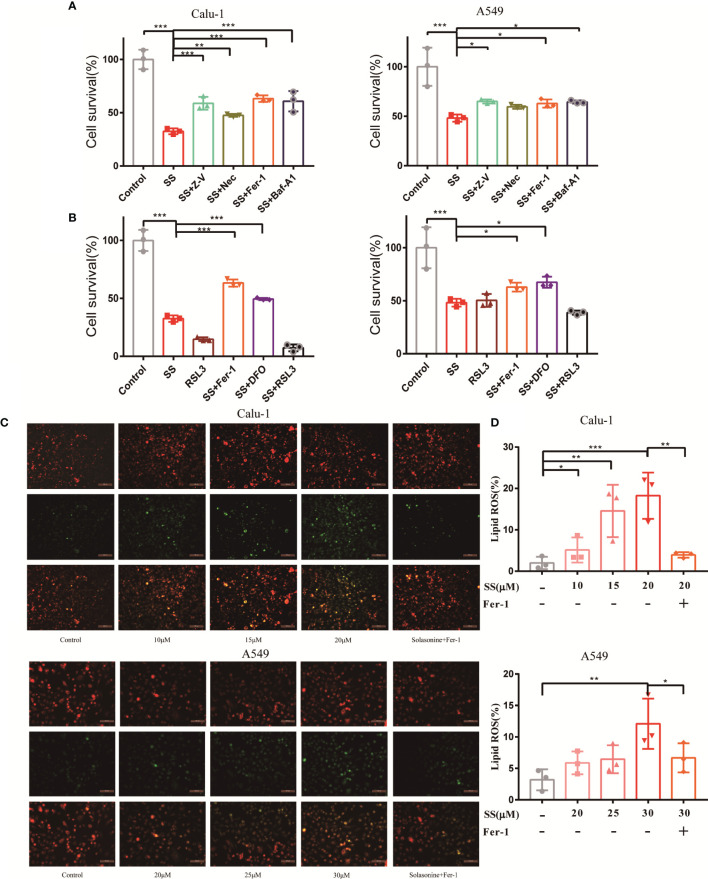
Ferroptosis contributed to solasonine-induced cell death in LUAD cells. **(A, B)** CCK8 assay was used to determine the cell viability of Calu-1 or A549 cells treated with 20 μM or 30 μM SS, respectively, with or without pretreatment of Z-V (20 μM), Nec (30 μM), Fer-1 (1μM), Baf-A1 (100 nM), RSL3 (0.5 μM), and DFO (80 μM). **(C)** Fluorescence microscopy was used to detect the lipid ROS in Calu-1 or A549 cells, which were respectively treated with 10, 15, 20 μM or 20, 25, 30 μM SS for 6 h and pretreated with or without Fer-1(1 μM). Scale bars: 100μm. **(D)** Flow cytometry was used to detect the double signals of C11-BODIPY581/591, which were then digitized and analyzed using histogram statistics. (*P < 0.05, **P < 0.01, ***P < 0.001).

What types of cell death contributed to the solasonine-induced growth inhibition in LUAD cells? This question prompted us to conduct additional research into the work. In the CCK8 assay, the descent was rescued by Z-V, Nec, Fer-1, and Baf-A1, indicating that apoptosis, necroptosis, ferroptosis, and autophagy may play a part in solasonine-induced cell death (**Figure 2A**). Several ferroptosis-related reagents, including inhibitor DFO and inducer RSL3, were used for extra validation to assess the involvement of ferroptosis. Fer-1, a lipid peroxidation inhibitor, and DFO, an iron chelator, are both commonly used ferroptosis inhibitors and are widely used in ferroptosis reports (13). In addition, since their discovery, RSL3 and erastin have been well known for their ferroptosis-induced property (14). The data indicated that Fer-1 and DFO protected both cells from solasonine-induced destruction, however, the toxicity was amplified by the combination of RSL3 and SS (**Figure 2B**). The fluorescent indicator C11-BODIPY581/591 was used to directly observe lipid peroxidation, which is located in the membrane and shifts the fluorescence from red to green when oxidization occurred (15). As expected, solasonine-treated Calu-1 and A549 cells exhibited high fluorescence in green and low fluorescence in red, referring to the accumulation of lipid peroxidation, whereas the Fer-1 restricted this process (**Figures 2C, D**).

### Solasonine Caused Iron Overload and Redox Imbalance in LUAD Cells

**Figure 3:**
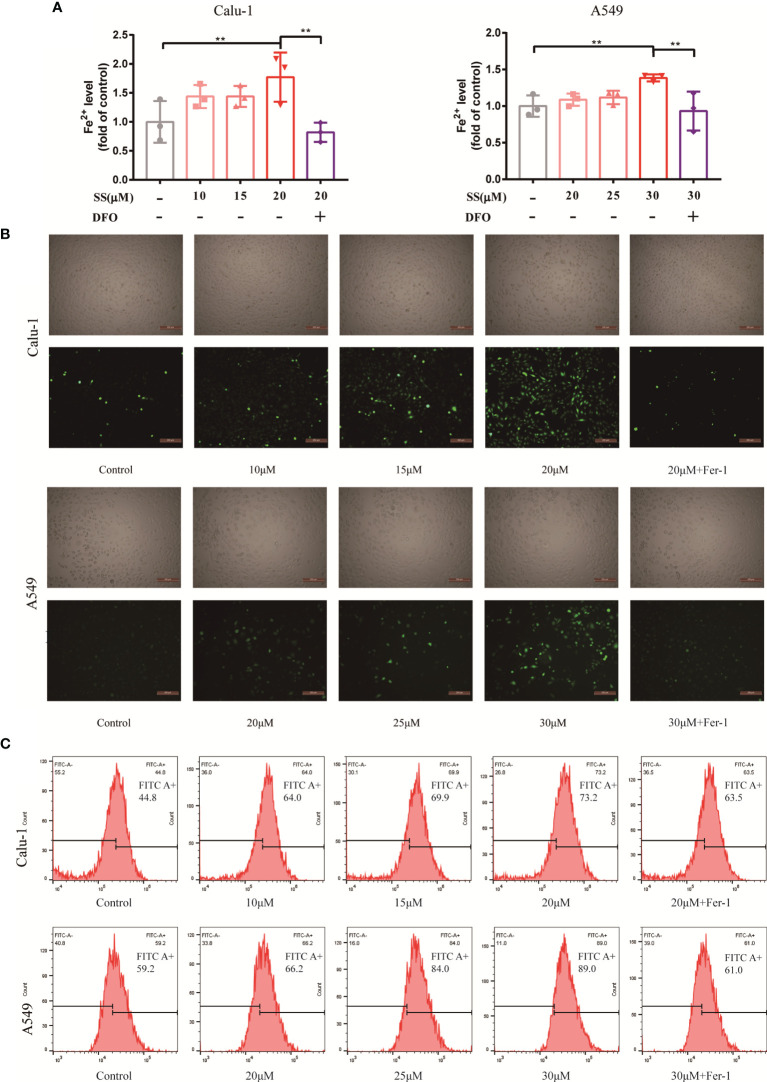
SS caused Fe^2+^ overload and ROS accumulation in LUAD cells. **(A)** Flow cytometry was used to detect the Fe^2+^ level in Calu-1 or A549 cells, which were respectively treated with 10, 15, 20 μM or 20, 25, 30 μM of SS for 6 h and pretreated with or without DFO (80 μM), the data statistic was shown in a histogram. (**P < 0.01). **(B, C)** Fluorescence microscopy and flow cytometry were used to detect ROS production in Calu-1 or A549 cells treated for 6 h with 10, 15, 20 μM or 20, 25, 30 μM SS and pretreated with or without Fer-1 (1 μM), scale bars: 200 μm.

Since ferroptosis has been identified, the critical events of the process, which include Fe2+overload and intracellular ROS accumulation, will be investigated and validated in the next step. It should be noted that the occurrence of ferroptosis is dependent on the level of iron content rather than any other metallic element, so measuring the content of Fe2+ is required. Fortunately, Fe2+ was discovered to be required for SS action, as evidenced by the experimental results shown in **Figure 3A**. The levels of Fe2+ increased as drug concentrations increased. However, a high dose of SS had a remarkable effect, which was completely reversed by DFO. On the other hand, intracellular ROS, while not as specific as lipid ROS in ferroptosis, is a major character that runs throughout the entire process. The images demonstrated that the intracellular ROS marked with DCFH-DA probes was excessively generated when SS was administered (**Figure 3B**). Besides the fluorescent images, the detailed information about the integral fluorescent intensity was presented in the histogram (**Figure 3C**).

### The Destruction of the Glutathione Redox System Caused by Solasonine Contributed to Ferroptosis in LUAD Cells

**Figure 4:**
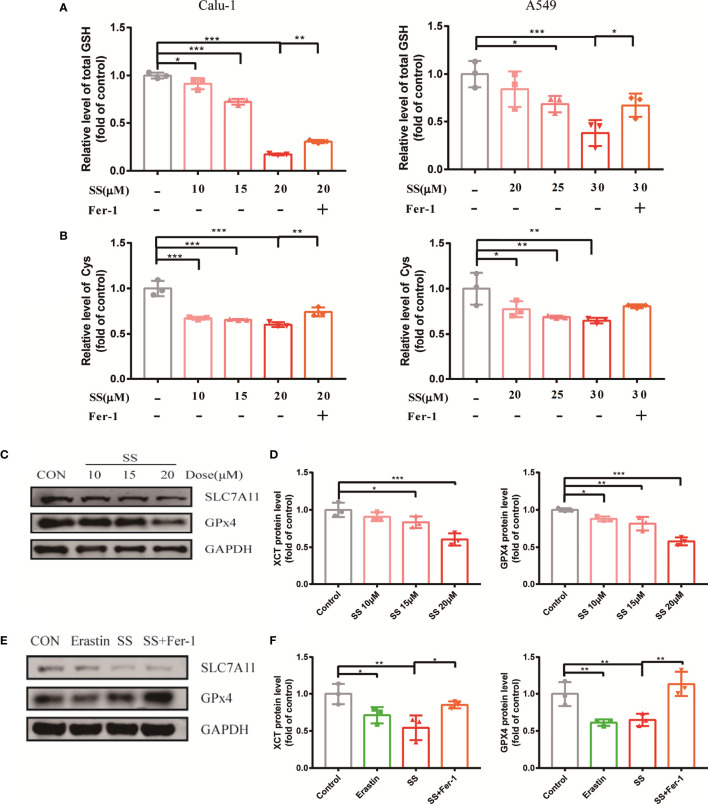
SS attenuated the oxidation resistance of LUAD cells. **(A, B)** GSH or Cys was detected in Calu-1 or A549 cells, which were respectively treated with 10, 15, 20 μM or 20, 25, 30 μM SS for 6 h, and pretreated with or without Fer-1 (1 μM), the data statistic was shown in a histogram (*P < 0.05, **P < 0.01, ***P < 0.001). **(C)** Western blot analysis was used to detect the expressions of SLC7A11 and GPX4 in Calu-1 cells, which were treated with 10, 15, 20 μM SS for 6 h. **(D)** Quantitative analysis of gray value of the SLC7A11 and GPX4 blots. **(E)** Western blotting analysis was used to detect the expressions of SLC7A11 and GPX4 in Calu-1 cells, which were treated with 20 μM SS or 4 μM erastin, with or without Fer-1 (1 μM) for 6 h. **(F)** Quantitative analysis of gray value of the SLC7A11 and GPX4 blots.

When discussing ferroptosis, the glutathione redox system is a popular point of penetration. Intracellular antioxidant enzyme glutathione peroxidase 4 (GPX4), cystine/glutamate transporter SLC7A11, substrate GSH, and Cys all contribute to the antioxidant machinery’s response to redox imbalance. The statistical graph displayed that the GSH and Cys decreased significantly when compared to the control group (**Figures 4A, B**).

Furthermore, the Western blot results revealed that the protein expression of GPX4 and SLC7A11 was altered on the sensitive cell line Calu-1 between SS treated cells and the control (**Figure 4C**), and the gray value was transformed into quantitative data displayed by the bar graph (**Figure 4D**). It is suggested that the inhibition of the expression of protein GPX4 and SLC7A11 by SS had parallels with the effect of the positive control group (**Figures 4E, F**). Incidentally, Fer-1 successfully counteracted the effect of SS on these four redox components to some extent. All the evidence pointed to SS paralyzing the antioxidant defense, collapsing the redox balance, and causing ferroptosis.

### Mitochondrial Injury Contributed to the Solasonine-Induced Ferroptosis in LUAD Cells

It is well known that the mitochondria are the cell’s energy centers, responding to various stimuli and conducting oxidative metabolism in some biological activities. Thus far, studies have shown that mitochondria, a major site for ROS production, play a critical role in ferroptosis (16). Therefore, the relationship between mitochondria and solasonine-induced ferroptosis on Calu-1 cells needs to be revealed.

**Figure 5:**
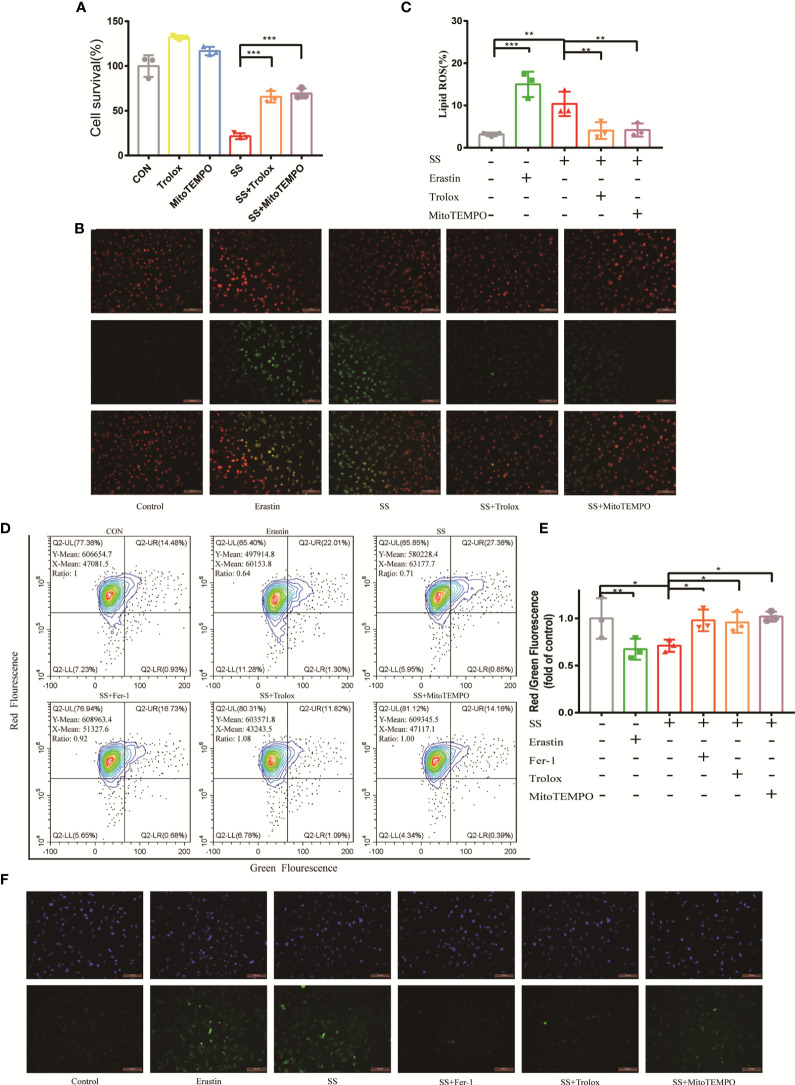
The dysfunction of mitochondria contributed to solasonine-induced ferroptosis in LUAD cells. **(A)** CCK8 assay was used to determine the cell viability of Calu-1,which were treated with 20 μM SS, and pretreated with or without Trolox (100 μM) or Mito TEMPO (10 μM). **(B)** Fluorescence microscopy was used to detect the lipid ROS production in Calu-1cells, which were treated with 20 μM SS for 6 h and pretreated with or without Trolox (100 μM) or Mito TEMPO (10 μM). Scale bars: 100 μm. **(C)** Flow cytometry was used to detect the double signals of C11-BODIPY581/591, which were then digitized and analyzed using histogram statistics (*P < 0.05, **P < 0.01, ***P < 0.001). **(D)** JC-1 staining was used to detect the mitochondrial transmembrane potential (Δψmt) in Calu-1 cells, which were treated with 20 μM SS or erastin (4 μM) for 6 h and pretreated with or without Fer-1 (1 μM), Trolox (100 μM), or Mito TEMPO (10 μM). **(E)** Quantitative analysis of red/green fluorescence ratio of cells. **(F)** Fluorescence microscopy was used to detect the lipid ROS of mitochondria in Calu-1cells, which were treated with 20 μM SS or erastin for 6 h and pretreated with or without Fer-1 (1 μM), Trolox (100 μM), or Mito TEMPO (10 μM). Scale bars: 100 μm. Nuclei were stained with Hoechst 3342. Scale bars: 100 μm.

To begin with, the viability of the cells was determined using the antioxidants Trolox and MitoTEMPO. Trolox, in addition to protecting cells from oxidation, prevents membrane damage for cell integrity, and MitoTEMPO targets superoxide and alkyl radicals found in mitochondria. With the assistance of Trolox and MitoTEMPO, the solasonine-treated cells rejuvenated to a great extent (**Figure 5A**).

According to these findings, mitochondria were found to play a role in solasonine-induced ferroptosis. To gain more insights, mitochondrial functional assessments such as mitochondrial injury and mitochondrial lipid peroxidation assays were launched. According to the diagram, the solasonine-treated group with a low fluorescent ratio showed high mitochondrial membrane potential depolarization paralleled to the positive control. However, this alternation could be reversed after using Fer-1, Trolox, and MitoTEMPO (**Figures 5D, E**). Moreover, the level of mitochondrial ROS production was evaluated using MitoPeDPP dye, a selective probe that targets peroxide in the mitochondrial inner membrane (17). The enhancement of a green, fluorescent signal indicated that erastin or SS action increased mitochondrial ROS production. In addition to Fer-1, Trolox, and MitoTEMPO, they were also capable of reversing excessive mitochondrial ROS production (**Figure 5F**). The data showed that the SS may obstruct mitochondrial function directly and exacerbate the redox imbalance when ferroptosis occurs on LUAC cells.

## Discussion

In addition to surgery, chemotherapy, and radiotherapy, common cancer treatment options include innovative targeted therapies that are aimed at selectively eliminating malignant cells to the greatest extent feasible, hopefully without damaging normal cells.

Because the RCD process involves several deadly subroutines that can intervene in tumor formation and progression, it has enabled breakthroughs in cancer treatment (18). To take advantage of this, ongoing research on apoptosis, necroptosis, pyroptosis, and ferroptosis in response to various malignancies is being conducted (19). With a better understanding of the specific molecular mechanism, ferroptosis is looking like a viable option.

Ferroptosis, in particular, is a critical impediment to LUAD development. The high ventilation and active gaseous metabolism of the lungs shape a unique condition in the tumor setting when compared to other tissues. Carcinogenic cells evolve sophisticated mechanisms to adapt to high-oxygen tension under high oxidative stress, indicating that these carcinogenic cells are sensitive to ferroptosis (20). Erastin was the first to trigger LUAD cell ferroptosis in K-ras mutant A549 cells (21). According to the following article, the combination of erastin and cisplatin had a synergistic impact in suppressing LUAD cells in a ferroptosis-like way by depleting GSH and inactivating GPXs (22).

The main character in the current study was the natural compound solasonine, which had an excellent tumor-suppression performance in LUAD, as shown in **Figure 1**. Zhang previously discovered that SS induced apoptosis and cell cycle arrest in acute monocytic leukemia through upregulating the AMPK/FOXO3A pathway (9). Wang claimed that SS inhibited glioma growth by modulating MAPK signaling *via* p-p38 and p-JNK in the inflammatory signaling pathway (10). Furthermore, SS was found to be effective against gastric cancer through modifying the miR-486-5p/PI3KR1 axis (11). What counts is that SS has been linked to tumor cell ferroptosis, which provides the potential that it may contribute to LUAD cell death *via* ferroptosis. Fortunately, observations of ferroptosis-related products and cell viability corroborated the theory.

Since the SS was shown to reduce cell viability, the cell-rescue would be observed by using numerous cell death inhibitors to determine the potential role of various cell death mechanisms. As shown in **Figure 2A**, ferroptosis is partially responsible for cell death. The effect of Fer-1 was similar to that of Z-V, Nec, and Baf-A1, suggesting that solasonine-induced cell death may be a synergistic result. This basic experiment provides a valuable reference in the preliminary stage of the study. Although ferroptosis appears to be independent of other known cell death mechanisms, there are some crossovers between ferroptosis and other cell death pathways. According to new data, ferroptosis is an autophagic cell death process that is aided by autophagy’s hyperactive lysosome activity, which regulates iron homeostasis and ferroptosis-associated ROS production (23, 24). In addition, P53, a canonical tumor suppressor protein, has been found to induce ferroptosis in certain conditions, suggesting molecular interaction between ferroptosis and apoptosis (25). Necroptosis and ferroptosis can be triggered by erastin simultaneously (3). The ferroptosis may affect or be influenced by other RCDs, specific efforts should be made in the future to demonstrate the relationship.

The findings illustrated that SS increased iron accumulation and lipid peroxidation, which could be limited by Fer-1, which is thought to cause ferroptosis in LUAD cells. Essentially, glutathione-dependent antioxidant defenses were the primary focus of research into the mechanism of ferroptosis. Inhibiting the cystine-glutamate antiporter (system Xc–) and the downstream enzyme glutathione peroxidase 4 (GPX4) appears to have depleted glutathione (GSH), a major cellular antioxidant whose synthesis requires Cys, resulting in antioxidant defense failure in ferroptosis (26, 27). SLC7A11 is a subunit of system Xc–, which was identified early on to be one of the most vital regulators in ferroptosis.

Because SLC7A11 is a recognized target in ferroptosis, blocking it will directly rein in the uptake of Cys, which can be utilized for GSH synthesis in the antioxidant process (28). Moreover, SLC7A11 has been found to be highly expressed in non-small cell lung cancer (NSCLC) with a poor prognosis (29). After that, the presence of GSH is required for the other canonical biomarker of ferroptosis, GPX4, to detoxify lipid hydroperoxides (30). It follows that these widely reported bio-markers of ferroptosis were actually affected by SS. Taken together, our findings (**Figure 4**) suggest that suppression of SLC711 and GPx4 expression was likely to be responsible for GSH and Cys depletion in SS therapy.

As our understanding of the mechanism of ferroptosis has progressed, mitochondria have been found to be associated with ferroptosis. Inhibiting the mitochondrial TCA cycle or the electron transfer chain (ETC) reduced hyperpolarization of the mitochondrial membrane potential, lipid peroxide accumulation, and ferroptosis (16, 31). The reaction between ROS and polyunsaturated fatty acids of lipid membranes can enhance lipid peroxidation that depended on the particular lipid precursor afforded by mitochondrial fatty-acid metabolism (32). Moreover, the previous research elaborated that peroxidized lipids were preferentially transferred to mitochondria by the mitochondrial membrane lipid transport protein and intercepting this process would mitigate the effect of ferroptosis (33). Nevertheless, the concrete contribution of mitochondria to ferroptosis may be context-dependent; to determine unambiguously whether mitochondria play a role in solasonine-induced ferroptosis, we set a succession of examinations with mitochondria.

Intriguingly, the cells exposed to SS showed the amplification of mitochondrial lipid ROS, suggesting the potential involvement of mitochondria in solasonine-induced ferroptosis (**Figure 5F**). A sequence of metabolic activities occurs across the mitochondrial membrane, and hyperpolarization of the mitochondrial membrane potential (MMP) has been observed in ferroptosis, which reflects the subsequent formation of lipid ROS (16). The JC-1 was used in this case to signify MMP in the process of accomplishing these tentative efforts. As shown in the image, MMP hyperpolarization was discovered, which was attributed to SS (**Figures 5D, E**). The high cell mortality, lipid ROS production, MMP hyperpolarization, and oxidative lipid in mitochondria were significantly alleviated with the antioxidant Trolox and mitochondrial ROS scavenger MitoTEMPO, highlighting the role of mitochondrial ROS in the SS treatment (**Figure 5**).

## Conclusions

To determine the medical properties of solasonine, this research used LUAD cells that were exposed to the compound, with the results indicating that the compound was able to inhibit LUAD cells and cause ferroptosis. As a result, it was concluded that redox imbalance and mitochondrial malfunction were the essential factors in solasonine ferroptosis. From an objective point of view, there were some unaddressed issues in this study, such as the precise targets and causative link of mitochondrial injury. It is obvious that the research will continue because the next stage will involve more in-depth studies. Incidentally, the findings of this study support previous research and serve as a benchmark for future research into solasonine-induced ferroptosis in the treatment of LUAD.

## Data Availability Statement

The raw data supporting the conclusions of this article will be made available by the authors, without undue reservation.

## Author Contributions

YL and J-CW contributed conception and design of the study. Y-YZ performed the experimental practices and wrote the original draft. Y-BL conducted the analytic process. BZ and Y-BP generated the figures. W-JT and Y-JC contributed to data sorting and literature search. J-HT performed the statistical analysis and analysis outcome. X-DJ reviewed and edited the manuscript. All authors contributed to the article and approved the submitted version.

## Funding

This research was funded by National Natural Science Foundation of China (81973795), National Natural Science Foundation of China (82174183), Shanghai Pujiang Program (2020PJD057) and Clinical Research Plan of SHDC (SHDC2020CR4052).

## Conflict of Interest

The authors declare that the research was conducted in the absence of any commercial or financial relationships that could be construed as a potential conflict of interest.

## Publisher’s Note

All claims expressed in this article are solely those of the authors and do not necessarily represent those of their affiliated organizations, or those of the publisher, the editors and the reviewers. Any product that may be evaluated in this article, or claim that may be made by its manufacturer, is not guaranteed or endorsed by the publisher.
